# FACILITATING CARE: A NARRATIVE APPROACH TO ASSESS EXPERIENCED QUALITY OF CARE

**DOI:** 10.1093/geroni/igz038.284

**Published:** 2019-11-08

**Authors:** Katya Sion, Hilde Verbeek, Gaby Odekerken-Schröder, Sandra Zwakhalen, Jos Schols, Jan Hamers

**Affiliations:** 1 Department of Health Services Research, Care and Public Health Research Institute (CAPHRI), Faculty of Health, Medicine and Life Sciences, Maastricht, Netherlands; 2 Department of Health Services Research, Care and Public Health Research Institute (CAPHRI), Faculty of Health, Medicine and Life Sciences, Maastricht, Limburg, Netherlands; 3 Department of Marketing and Supply Chain Management, Maastricht, Limburg, Netherlands

## Abstract

This study aimed to develop a method to assess experienced quality of care (QoC) in nursing homes from the resident’s perspective. A narrative approach “Facilitating Care” (FC) was developed based on the INDEXQUAL framework of experienced QoC and a needs assessment. FC assesses experienced QoC by training care professionals to perform individual conversations with residents, their family and their professional caregivers (triads) in another organization than where they are employed. FC consists of three phases: 1) training, 2) data collection and registration, and 3) analysis and reporting of the results. In 2018, 16 care professionals were trained and performed 148 conversations (47 residents, 44 family members, 57 professional caregivers) in 8 different nursing homes. Evaluation showed that FC teaches helpful conversation techniques and provides valuable insights into residents’ experienced QoC. Whilst the process was considered time consuming, all participants emphasized the added value of taking time for FC conversations.

